# Spatial genetic structure of *Stryphnodendron adstringens* (MART.) COVILLE (*Leguminosae-Mimosoideae*) using SSR markers

**DOI:** 10.1186/1753-6561-5-S7-P21

**Published:** 2011-09-13

**Authors:** Léo Zimback, Edson Mori, Eliete Branco, Adriano Lima, Eliseu Baena

**Affiliations:** 1 Instituto Florestal, Avaré, SP, 18701-180, Brazil; 2 Unesp, Botucatu, SP, 18610-307, Brazil; 3 University Sagrado Coração, Bauru, SP, 17011-160, Brazil

## Background

The forest fragments of *Stryphnodrendron adstringens* have disappeared because of the high rate of Brazilian Savannah deforestation for commercial purposes, such as eucalypt, citrus, soy bean, and sugar cane crops that have been intensely cultivated on those areas [1]. Economically, *S. adstringens* is important for manufacture industries of paints and tanneries, but also is utilized for therapy against ulcer, scurvy, and for antiseptic and healing purposes [2]. Considering the forestry sector, the species is potential for building uses because of their wood have high density and durability [3]. Aiming for germplasm preservation and for breeding programs we studied the spatial genetic structure of *S. adstringens* based on SSR markers.

## Methods

Juvenile leaves of 38 individuals of *S. adstringens* were collected in natural population, of approximately 200 individuals, in Botucatu State Forest belonging to Forestry Institute, São Paulo State, Brazil. The population is located from 22° 55' 55" S to 22° 56' 39" S latitude and from 48° 27' 19" W to 48° 27' 33" W longitude, through 860 m altitude, totalizing 33 hectares of savannah. The lab procedure protocol based on Ferreira and Grattapaglia [4], and adapted to *S. adstringens*, for DNA extraction was used. We assessed the transferability of 98 primer pairs developed for different tree species of several families and genera. The amplification was performed at 92° C for 2 minutes by initial denaturation, 45 cycles of 1 minute at 92° C, 1 minute at annealing temperature, and 1 min at 72° C, and ending to 10 minutes at 72° C. The analysis of spatial autocorrelation of sampled population, with local coordinates, and variable alleles of individuals was estimated. The "Spatial Genetic Software (SGS) version 1.0 program for distance classes per each allele, and considering 1000 bootstraps to get the Moran's I Index [5] were used. The expression was:

where:

I = Moran's I index, which can take values between -1.0 (negative autocorrelation) and + 1.0 (positive autocorrelation);

n = number of individuals;

pi and pj = allele frequency for i and j individuals;

p = average of p;

Wij = element of symmetric matrix square and W were nxn dimensions, which is given the value 1 for individual neighbors and 0 otherwise;

W = matrix that expresses the spatial correlation between individuals and the sum over i and j is the value iqual W.

## Results and discussion

Forty eight out of 98 pairs were amplified showing 23 polymorphic primers, but only 10 of them were conserved, and presenting no null alleles: EMBRA 03, EMBRA 06 (*Eucalyptus grandis*), EMBRA 72, EMBRA 210 (*Eugenia dysenterica*) Empas 02 (*Prunus avium*) LMCH 12, LMCH 14 (*Annona cherimoya*), SCU 056, SCU 062 (*Melaleuca alternifolia*), and SP 06 (*Schizolobium parahyba*). O LMCH 12 locus was eliminated to present low accuracy. Table 1 shows the correlogram of *S. adstringens* indicating that the distances, among individuals of Botucatu State Forest, from 0 to 33 and from 99 to 132 meters have presented spatial structure. According to Sebbenn [6]is common in tropical species, similarities until 100 meters; therefore to collect high variability of *S. adstringens* it is important obeyed distance up to 132 meters.

**Table 1:** Correlogram using Moran's Index (D) with confidence interval (CI), probability of exclusion (P), number of comparisons (CN), and value indicating absence of spatial autocorrelation D = -0.0270 for *Stryphnodendron adstringens*.

Distance (m)	D(-CI)	D(obs.)	D(+CI)	P(D)<(-CI)	P(D)>(+ CI)	CN
0-33	-0.080841	0.043351	0.034583	0.015	0.985	40
33-66	-0.078096	-0.005722	0.023789	0.190	0810	45
66-99	-0.079640	-0.010610	0.031820	0.291	0.709	41
99-132	-0.081936	-0.096614	0.032199	0.995	0.005	35
132-165	-0.079713	-0.015566	0.027000	0.355	0.645	38
165-198	-0.090142	0.025456	0.033938	0.046	0.954	37
198-231	-0.064909	-0.059062	0.007197	0.954	0.046	75
231-264	-0.086119	-0.063560	0.031723	0.867	0.133	25
264-297	-0.114183	-0.054740	0.074730	0.715	0.285	7
297-330	-0.103925	-0.058399	0.052559	0.800	0.200	10

## Conclusions

Significant results were observed for the class distances from 0 to 33 meters and 99 to 132 meters, showing there is spatial autocorrelation for *S. adstringens*.
